# Supplementary material for: Neural similarity predicts whether strangers become friends
Source: Nat Hum Behav. 2025 Aug 4;9(11):2285–98. doi: 10.1038/s41562-025-02266-7 (PMC12634445; doi:10.1038/s41562-025-02266-7)
Supplement: Supplementary file 1 — Supplementary Figs. 1–14, Tables 1–5, material on recruitment and data collection, and analyses using linear mixed models rather than node-level permutation testing. [file 41562_2025_2266_MOESM1_ESM.pdf]

nature human behaviour

Supplementary information https://doi.org/10.1038/s41562-025-02266-7

Neural similarity predicts whether strangers become friends

In the format provided by the authors and unedited

Supplementary Information

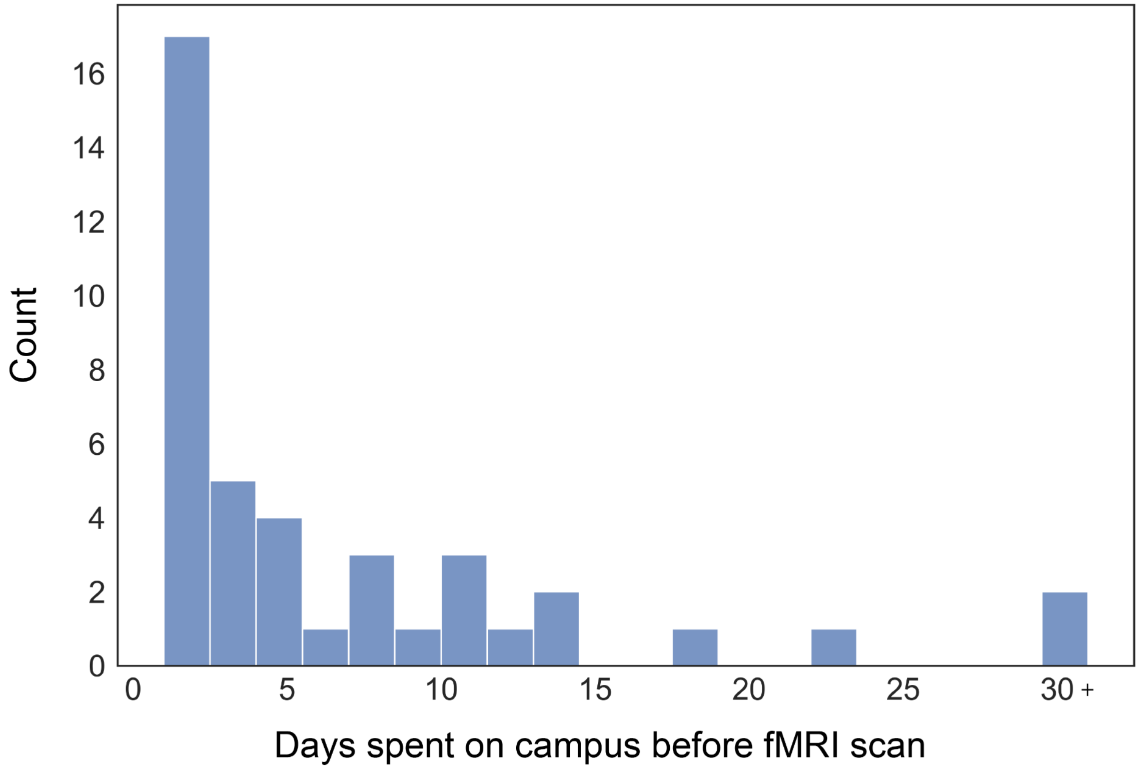

Supplementary Figure 1. Distribution of days between participants' arrival on campus and the fMRI study. The majority of fMRI participants were scanned shortly after their arrival on campus (Mode = 1 day; Median = 3 days). Only two participants were on campus for more than 30 days before the fMRI study; one participant was on campus for 400 days due to being enrolled in a prior graduate program at the same university, and the other participant was on campus for 1095 days due to being enrolled in a different prior graduate program at the same university. Thus, it was highly unlikely that participants formed meaningful relationships prior to the fMRI study. Additionally, we observed a similar pattern of results in the current study when excluding fMRI dyads who had encountered each other prior to the fMRI study (Supplementary Figures 5 and 9).

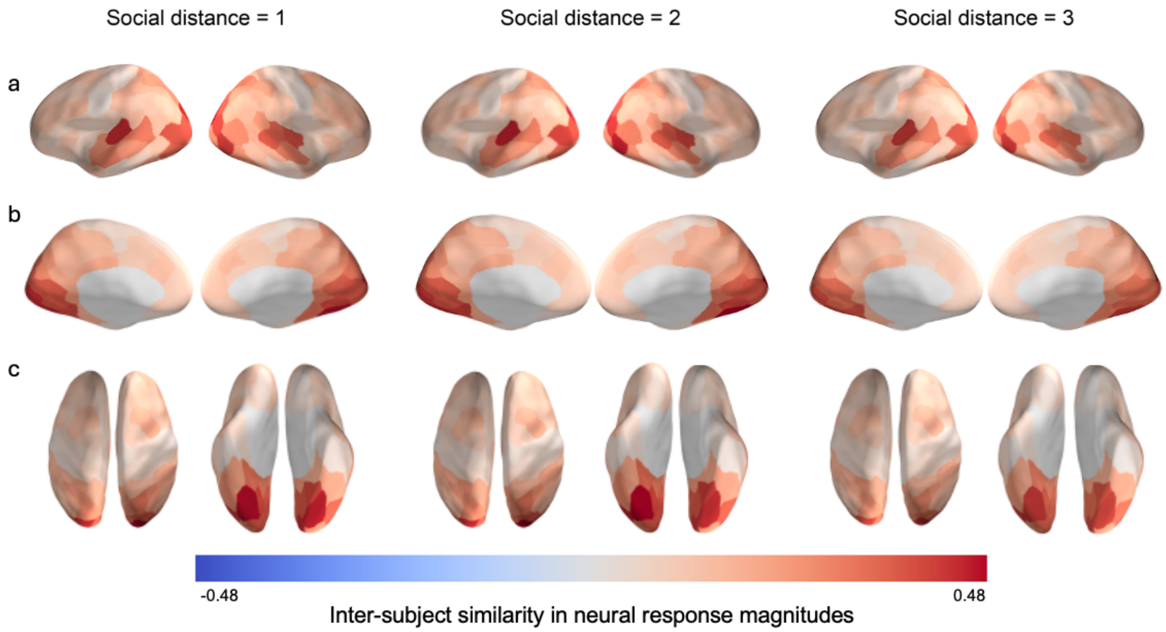

Supplementary Figure 2. Pre-existing neural similarities (not normalized within brain regions) averaged within levels of social distance measured 8 months later. Social distance, ranging from 1 to 3, is the geodesic distance between a pair of individuals in the social network characterized by reciprocal friendship ties. Data are overlaid on a cortical surface model and are shown in **(a)** lateral, **(b)** medial, and **(c)** dorsal and ventral views. Because these images merely depict the relative mean similarities for each brain region for each group of dyads, rather than the results of statistical tests, no thresholding has been performed. Inter-participant neural similarities were averaged within social distance level and then projected onto an inflated model of the cortical surface.

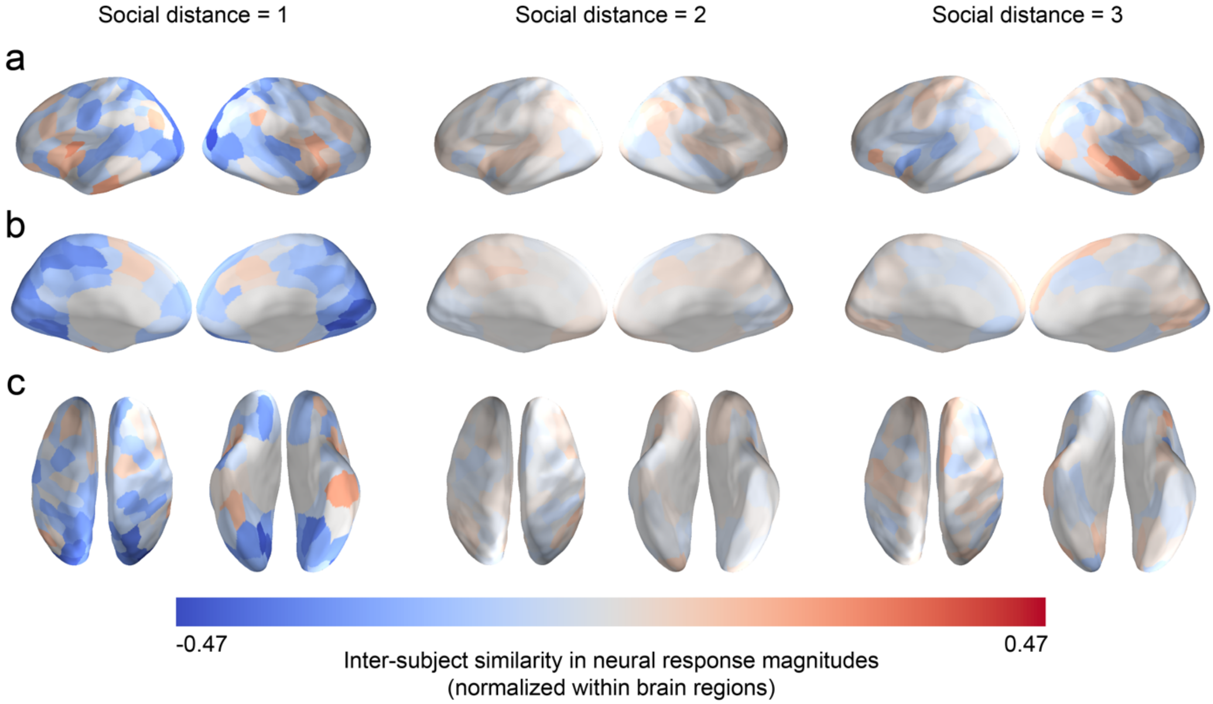

Supplementary Figure 3. Pre-existing neural similarities averaged within levels of social distance measured 2 months later at Time 2. Data are overlaid on a cortical surface model and are shown in **(a)** lateral, **(b)** medial, and **(c)** dorsal and ventral views. Because these images merely depict the relative mean similarities for each brain region for each group of dyads, rather than the results of statistical tests, no thresholding has been performed. Inter-participant neural similarities were normalized (i.e., z-scored across dyads for each region), averaged within social distance level, then projected onto an inflated model of the cortical surface. Warmer colors correspond to relatively similar neural responses for a given region, and cooler colors correspond to relatively dissimilar neural responses for a given region.

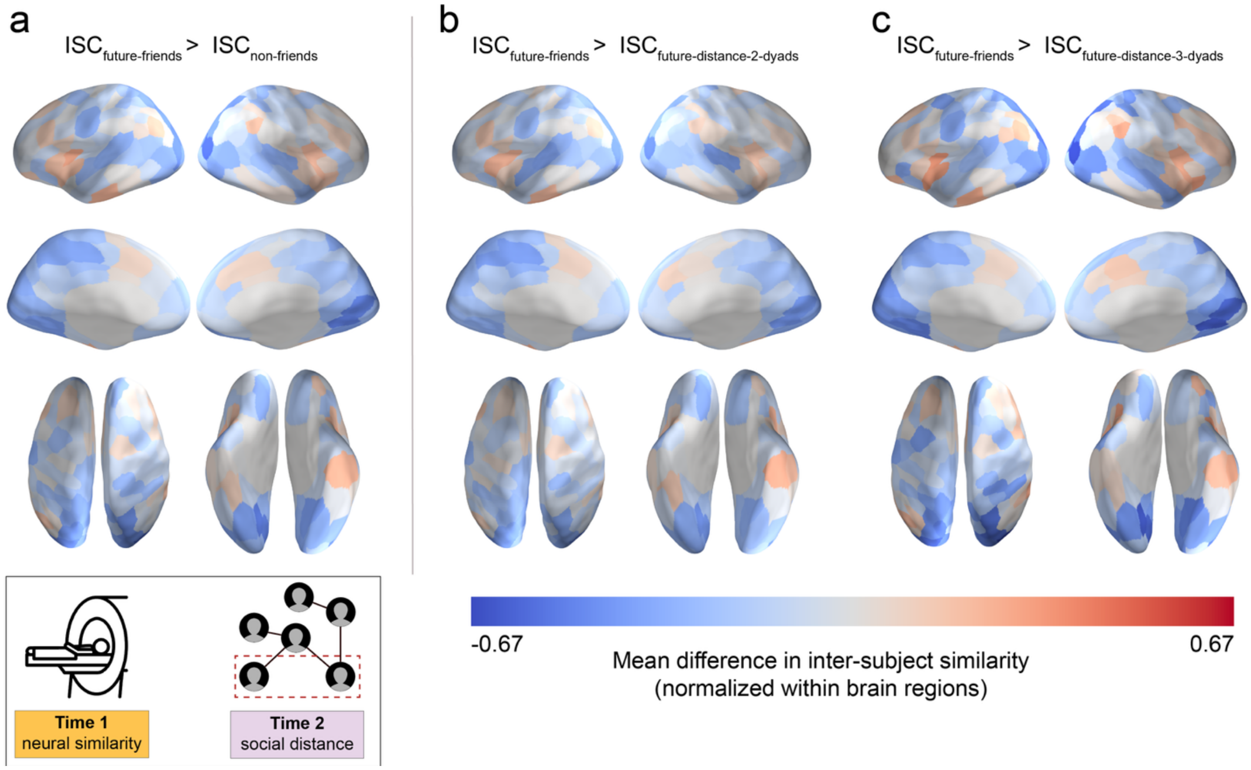

Supplementary Figure 4. Early in the school year, pre-existing neural similarities were not yet associated with friendship. Data are overlaid on a cortical surface model. The overlaid data is unthresholded. Warmer colors correspond to relatively greater mean neural similarity, and cooler colors correspond to relatively less mean neural similarity. Early in the school year (two months in, at Time 2), null results (statistical significance was determined using permutation testing) were observed when testing **(a)** if individuals who became friends exhibited greater pre-existing neural similarity relative to individuals who did not become friends and if individuals who became friends exhibited greater pre-existing neural similarity relative to **(b)** individuals who ended up in 2 degrees of separation from each other and **(c)** individuals who ended up in 3 degrees of separation from each other.

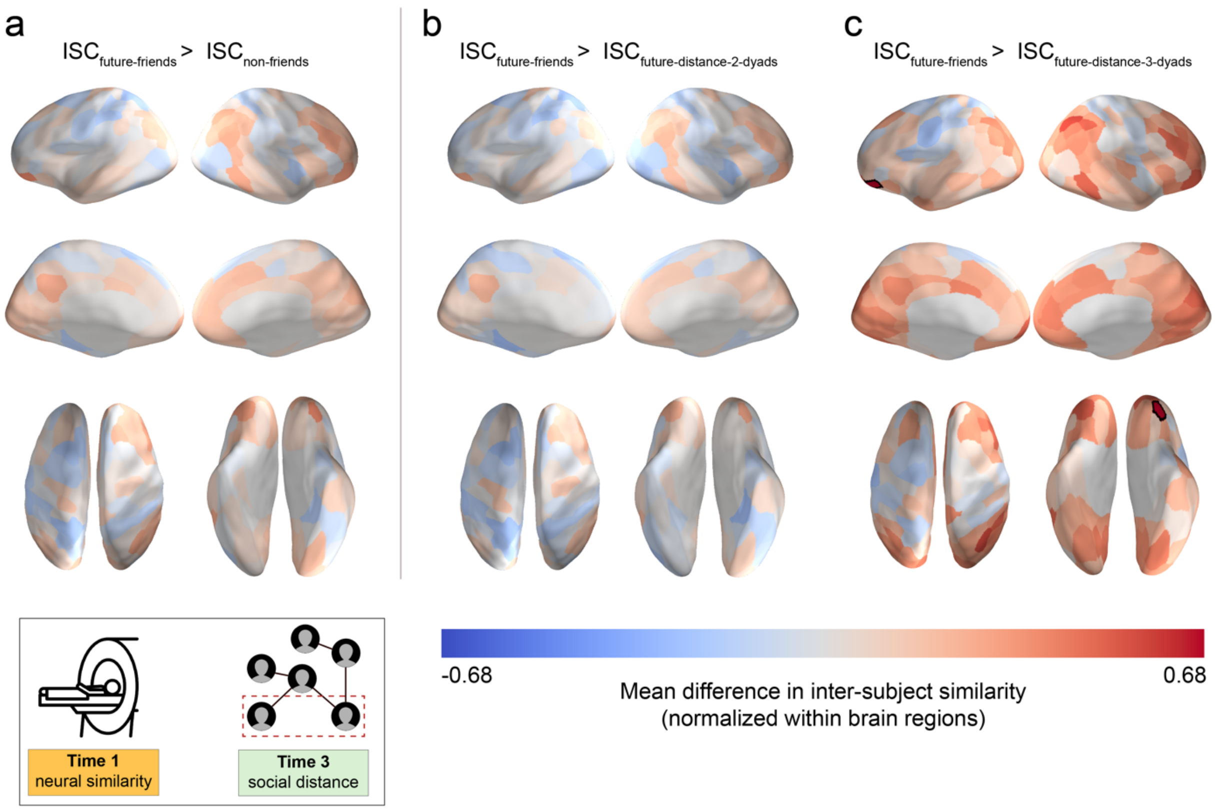

Supplementary Figure 5. People who became friends showed greater pre-existing neural similarity than those who ended up 3 "degrees of separation" from each other 8 months later when excluding dyads who met each other prior to the neuroimaging session. Data are overlaid on a cortical surface model. Warmer colors correspond to relatively greater mean neural similarity, and cooler colors correspond to relatively less mean neural similarity. Null results were observed when testing if individuals who became friends exhibited greater pre-existing neural similarity relative to **(a)** individuals who did not become friends and **(b)** individuals who ended up 2 degrees of separation from each other. **(c)** Individuals who became friends with each other showed greater pre-existing neural similarity in a portion of the left OFC ($p < 0.001$, FDR-corrected; statistical significance was determined using permutation testing) relative to individuals who ended up 3 degrees of separation from each other in the social network. The overlaid data is unthresholded; regions with significant differences, after FDR correction for multiple tests, for each contrast are outlined in black.

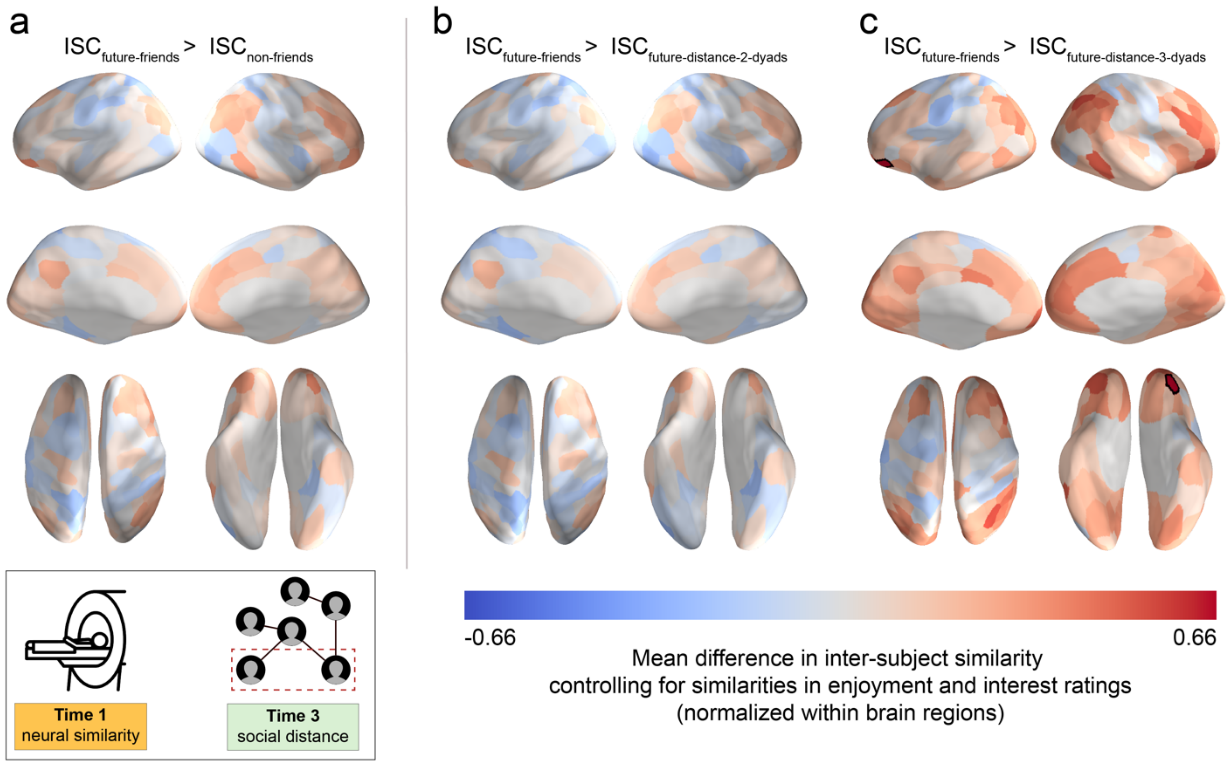

Supplementary Figure 6. People who became friends showed greater pre-existing neural similarity than those who ended up 3 "degrees of separation" from each other 8 months later when controlling for similarities in enjoyment and interest ratings. Data are overlaid on a cortical surface model. Warmer colors correspond to relatively greater mean neural similarity, and cooler colors correspond to relatively less mean neural similarity. (a) Null results were observed when testing if individuals who became friends exhibited greater pre-existing neural similarity relative to (b) individuals who did not become friends and (b) individuals who ended up 2 degrees of separation from each other. (c) Relative to individuals who ended up 3 degrees of separation from each other in the social network, individuals who became friends with each other showed greater pre-existing neural similarity in a portion of the left OFC ($p < 0.001$, FDR-corrected; statistical significance was determined using permutation testing). The overlaid data is unthresholded; regions with significant differences, after FDR correction for multiple tests, for each contrast are outlined in black.

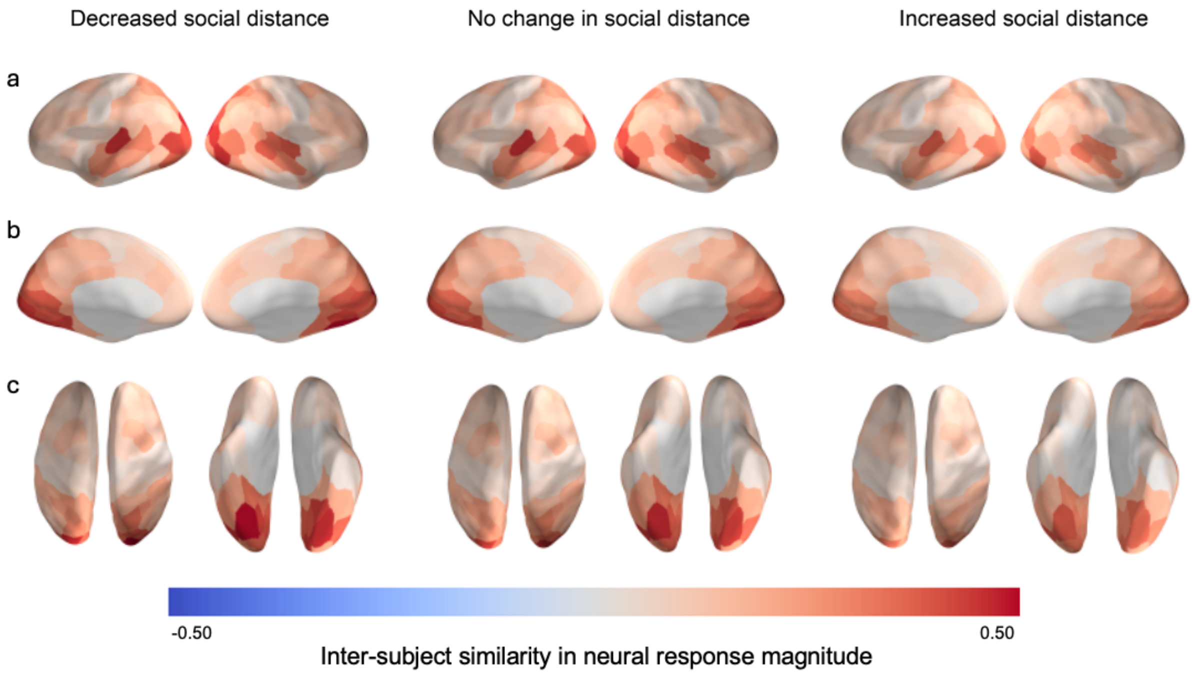

Supplementary Figure 7. Pre-existing inter-participant similarities (not normalized within brain regions) in mean neural response time series averaged within directions of change in social distance over time. Data are overlaid on a cortical surface model and are shown in **(a)** lateral, **(b)** medial, and **(c)** dorsal and ventral views. Because these images merely depict the relative mean similarities for each brain region for each group of dyads, rather than the results of statistical tests, no thresholding has been performed. Inter-participant neural similarities were averaged within directions of change in social distance and then projected onto an inflated model of the cortical surface.

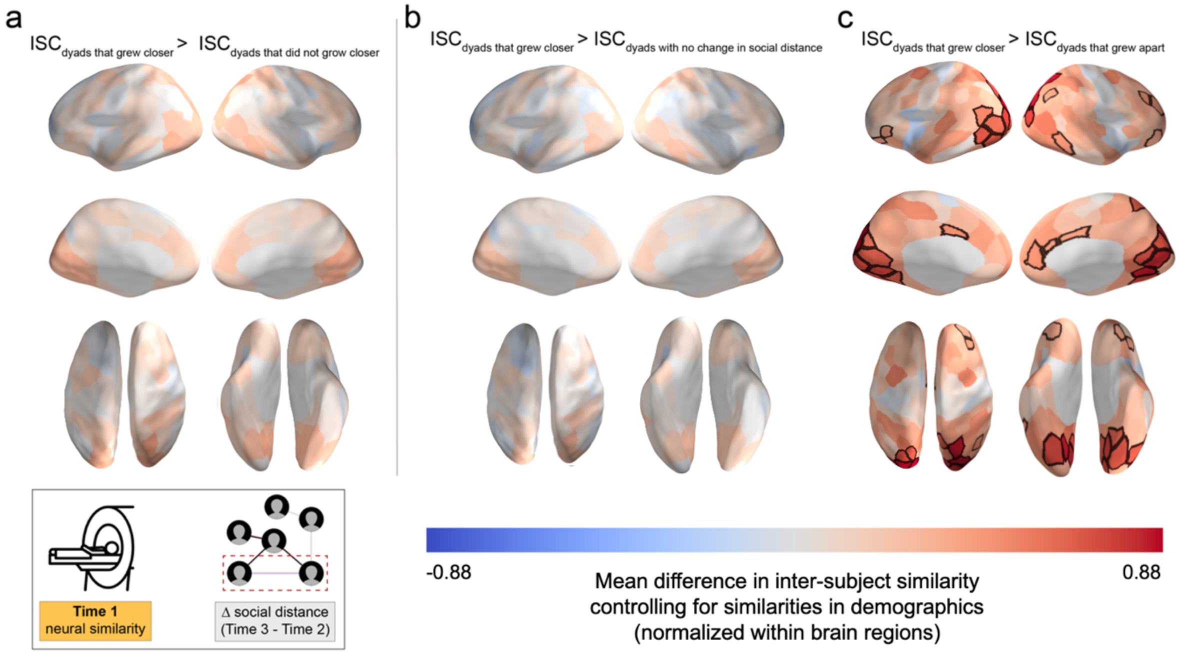

Supplementary Figure 8. Individuals who grew closer over time showed greater pre-existing similarity than individuals who grew farther apart over time when controlling for similarities in demographics (i.e., age, gender, nationality, hometown size and location, undergraduate alma mater location and institution type, undergraduate major, and industry). Data are overlaid on a cortical surface model. Warmer colors correspond to relatively greater mean neural similarity, and cooler colors correspond to relatively less mean neural similarity. For a given dyad, change in social distance over time was calculated by subtracting their social distance at Time 2 from their social distance at Time 3. Depending on these values, dyads were then placed into three categories depending on if their social distance increased, decreased, or remained the same (see Methods). Dyads whose social distance either increased over time or did not change were treated as a single category (i.e., dyads who did not grow closer). Individuals who grew closer over time (i.e., characterized by a decrease in social distance over time) did not exhibit significantly greater pre-existing neural similarity relative to **(a)** individuals who did not grow closer over time or **(b)** individuals whose social distance did not change. **(c)** Individuals who grew closer over time exhibited greater pre-existing neural similarity in regions spanning visual cortex, ventral temporal cortex, occipitotemporal cortex, superior parietal cortex, angular gyrus, medial frontal cortex, and lateral prefrontal cortex ($p < 0.05$, FDR-corrected; statistical significance was determined using permutation testing; *p*-values for each brain region that showed a significant effect were reported in Supplementary Table 3) relative to individuals who grew apart over time. The overlaid data is unthresholded, regions with significant differences, after FDR correction for multiple tests, for each contrast are outlined in black.

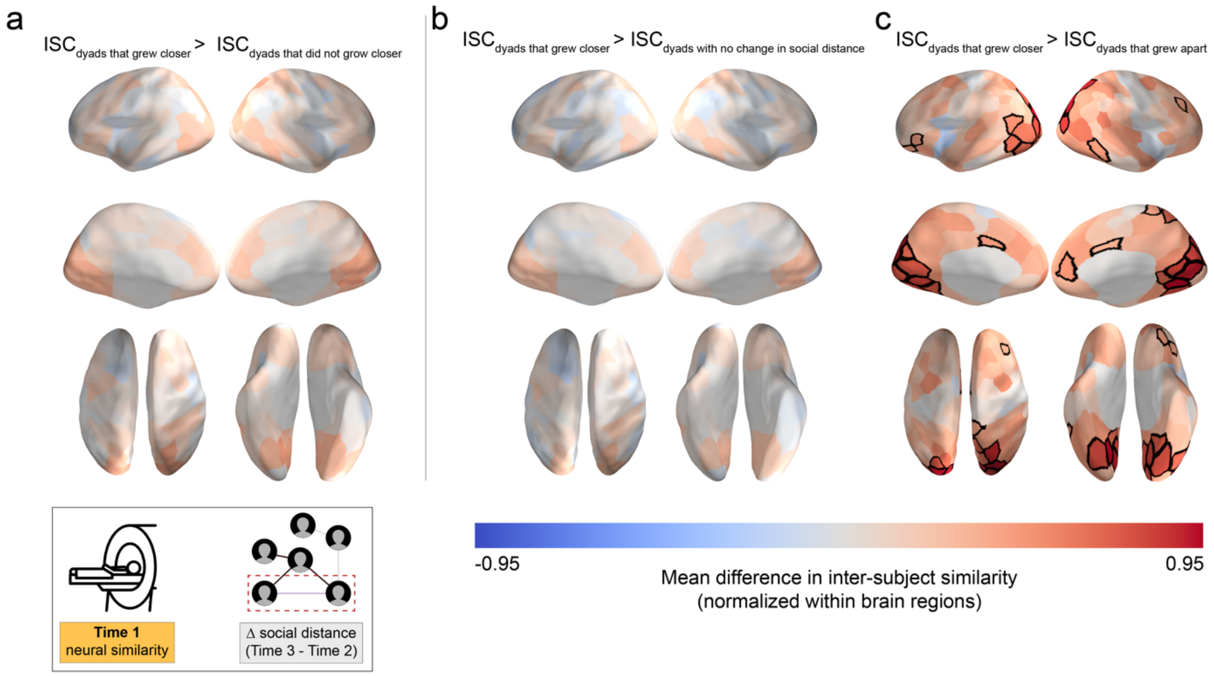

Supplementary Figure 9. Individuals who grew closer over time showed greater pre-existing similarity than individuals who grew farther apart over time when excluding dyads who knew each other prior to the neuroimaging session. Data are overlaid on a cortical surface model. Warmer colors correspond to relatively greater mean neural similarity, and cooler colors correspond to relatively less mean neural similarity. For a given dyad, change in social distance over time was calculated by subtracting their social distance at Time 2 from their social distance at Time 3. Depending on these values, dyads were then placed into three categories depending on if their social distance increased, decreased, or remained the same (see Methods). Dyads whose social distance either increased over time or did not change were treated as a single category (i.e., dyads who did not grow closer). Individuals who grew closer over time (i.e., characterized by a decrease in social distance over time) did not exhibit significantly greater pre-existing neural similarity relative to individuals **(a)** who did not grow closer over time or **(b)** whose social distance did not change. **(c)** Individuals who grew closer over time exhibited greater pre-existing neural similarity in regions spanning visual cortex, ventral temporal cortex, occipitotemporal cortex, superior parietal cortex, angular gyrus, medial frontal cortex, and lateral prefrontal cortex ($p < 0.05$, FDR-corrected; statistical significance was determined using permutation testing; p-values for each brain region that showed a significant effect were reported in Supplementary Table 4) relative to individuals who grew apart over time. The overlaid data is unthresholded; regions with significant differences, after FDR correction for multiple tests, for each contrast are outlined in black.

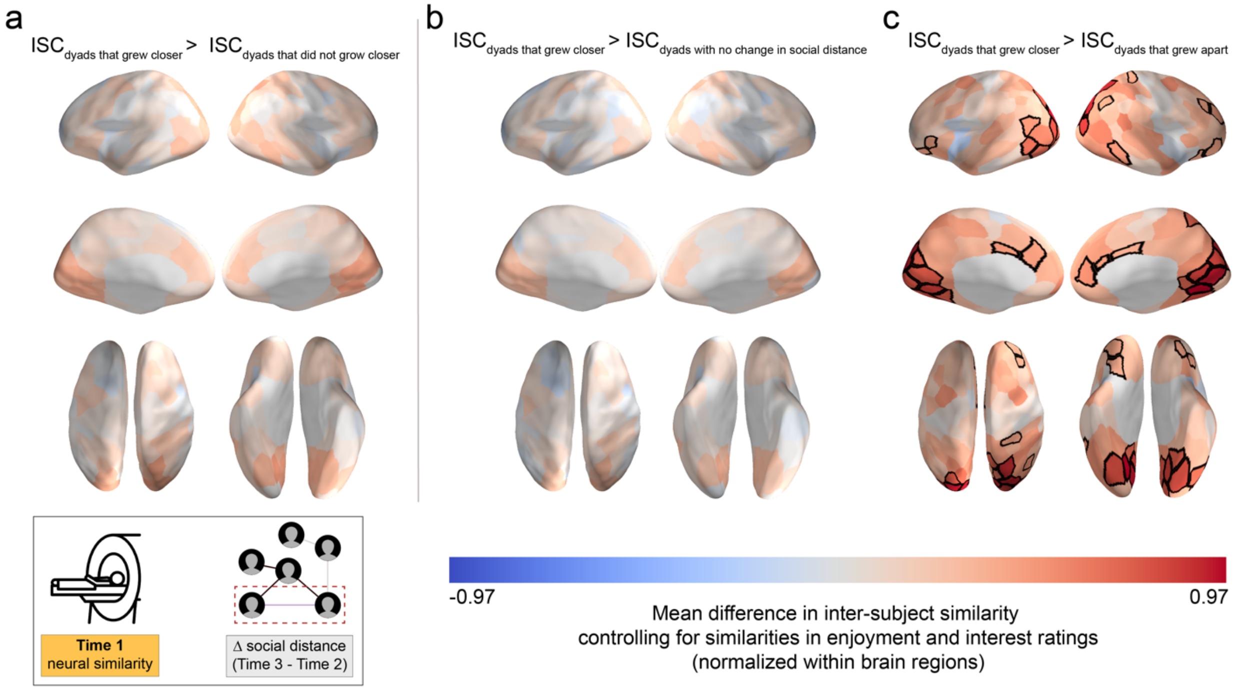

Supplementary Figure 10. Individuals who grew closer over time showed greater pre-existing similarity than individuals who grew farther apart over time when controlling for similarities in enjoyment and interest ratings. Data are overlaid on a cortical surface model. Warmer colors correspond to relatively greater mean neural similarity, and cooler colors correspond to relatively less mean neural similarity. For a given dyad, change in social distance over time was calculated by subtracting their social distance at Time 2 from their social distance at Time 3. Depending on these values, dyads were then placed into three categories depending on if their social distance increased, decreased, or remained the same (see Methods). Dyads whose social distance either increased over time or did not change were treated as a single category (i.e., dyads who did not grow closer). Individuals who grew closer over time (i.e., characterized by a decrease in social distance over time) did not exhibit significantly greater pre-existing neural similarity relative to **(a)** individuals who did not grow closer over time or **(b)** individuals whose social distance did not change. **(c)** Individuals who grew closer over time exhibited greater pre-existing neural similarity in regions spanning visual cortex, ventral temporal cortex, occipitotemporal cortex, superior parietal cortex, angular gyrus, medial frontal cortex, and lateral prefrontal cortex ($p < 0.05$, FDR-corrected; statistical significance was determined using permutation testing; p-values for each brain region that showed a significant effect were reported in Supplementary Table 5) relative to individuals who grew apart over time. The overlaid data is unthresholded; regions with significant differences, after FDR correction for multiple tests, for each contrast are outlined in black.

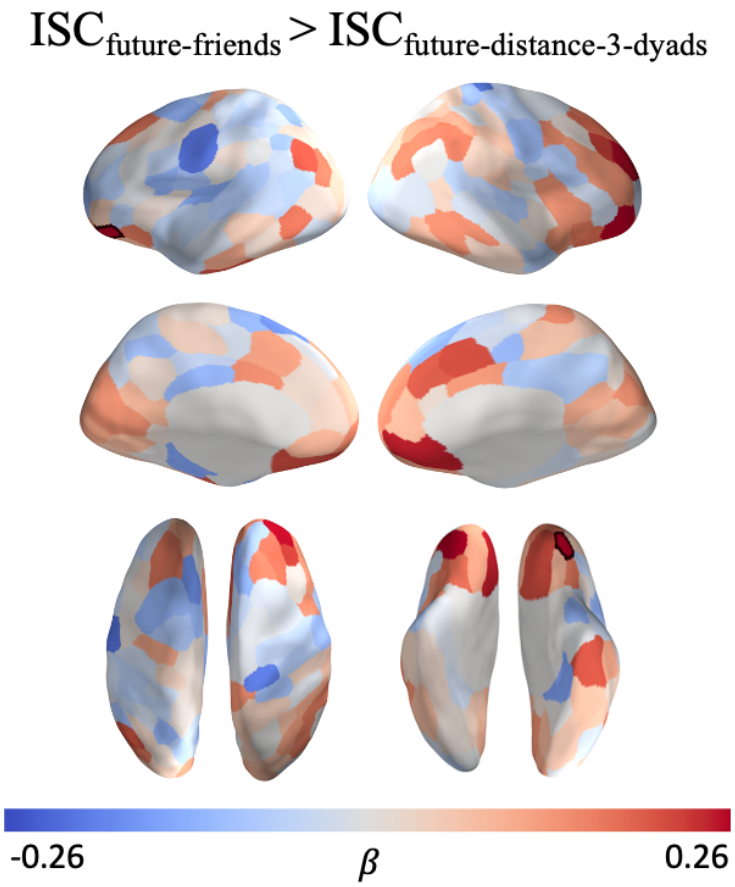

Supplementary Figure 11. Dyads who became friends with each other 8 months later showed greater pre-existing neural similarity than those who ended up with a social distance of 3 from each other. Data are overlaid on a cortical surface model. Warmer colors correspond to relatively greater neural similarity for future friends compared to other pairs of participants, and cooler colors correspond to relatively less neural similarity for future friends compared to other pairs of participants. Analysis using a linear mixed model showed that, aligning with results from node-permutation testing, individuals who became friends with each other showed greater pre-existing neural similarity in a portion of the left OFC ($\beta = 0.47$, SE = 0.12, $p < 0.001$, FDR-corrected; statistical significance was determined using permutation testing) relative to individuals who ended up 3 degrees of separation from each other in the social network 8 months after Time 1. Regions with the significant standardized regression coefficients, after FDR correction for multiple tests, are outlined in black.

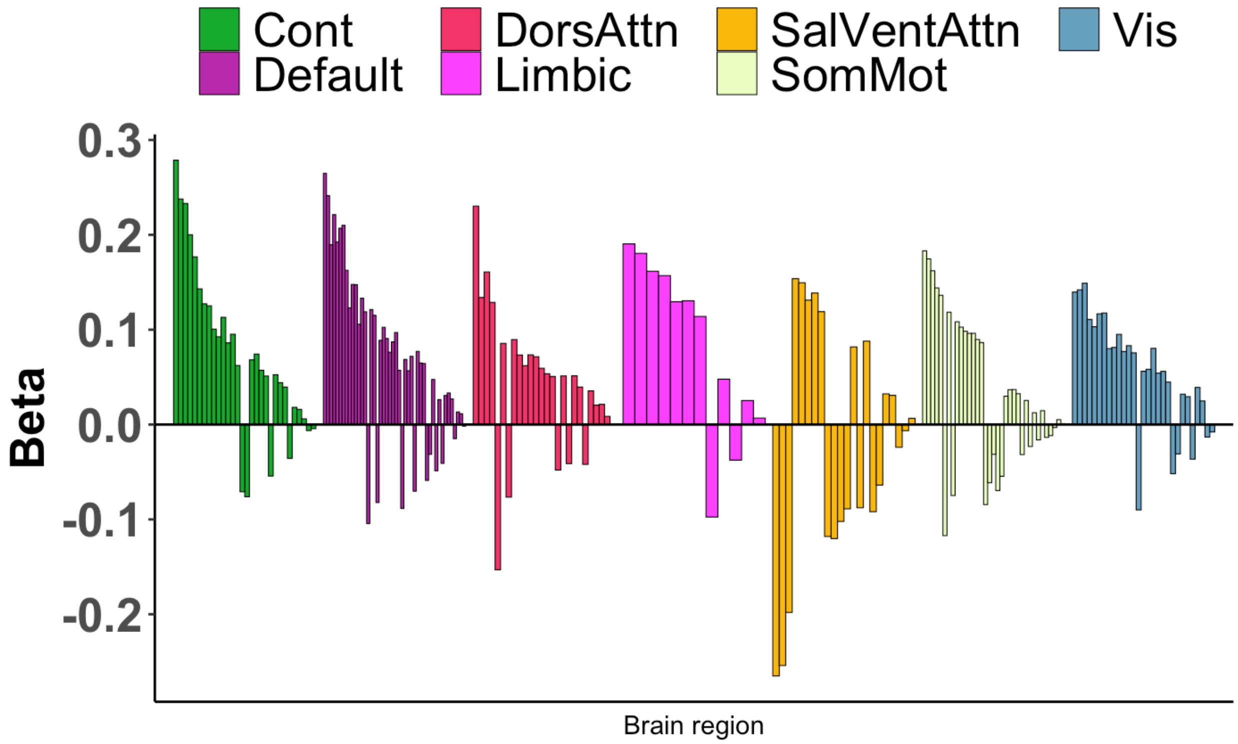

Supplementary Figure 12. Dyads that grew closer, relative to dyads that grew apart, tended to show higher neural similarity across brain regions spanning multiple brain networks. Regression coefficients for each brain parcel from the linear mixed model and planned contrast analysis for $ISC_{\text{dyads that grew closer}}$ vs $ISC_{\text{dyads that grew apart}}$, grouped by their corresponding brain networks, are shown.

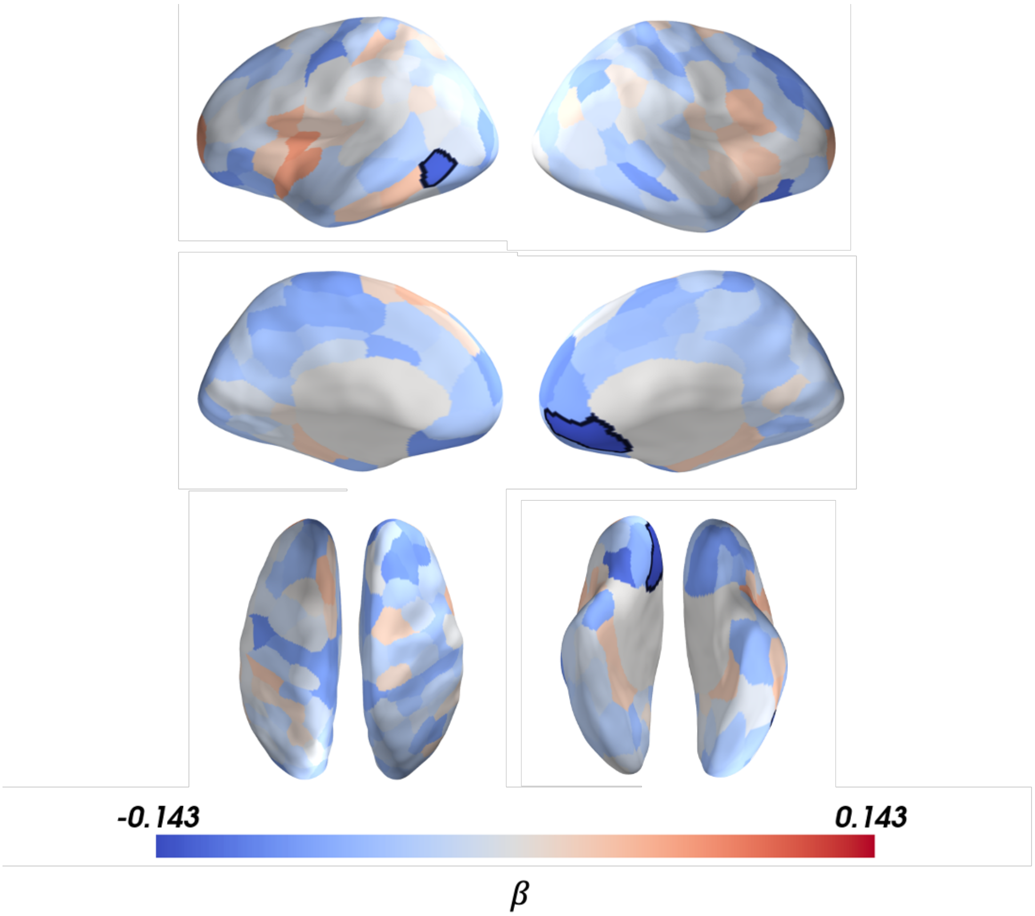

Supplementary Figure 13. Pre-existing neural similarity in regions of the ventromedial prefrontal cortex and lateral temporal cortex predicted decreases in social distance over time. There existed a trending negative association between pre-existing neural similarity and continuous change in social distance across brain regions. β is the standardized regression coefficient from the linear mixed effect model with crossed random effects for participants. Regions outlined in black demonstrated where significant associations ($p < 0.05$, FDR-corrected) were observed between continuous change in social distance and ISC.

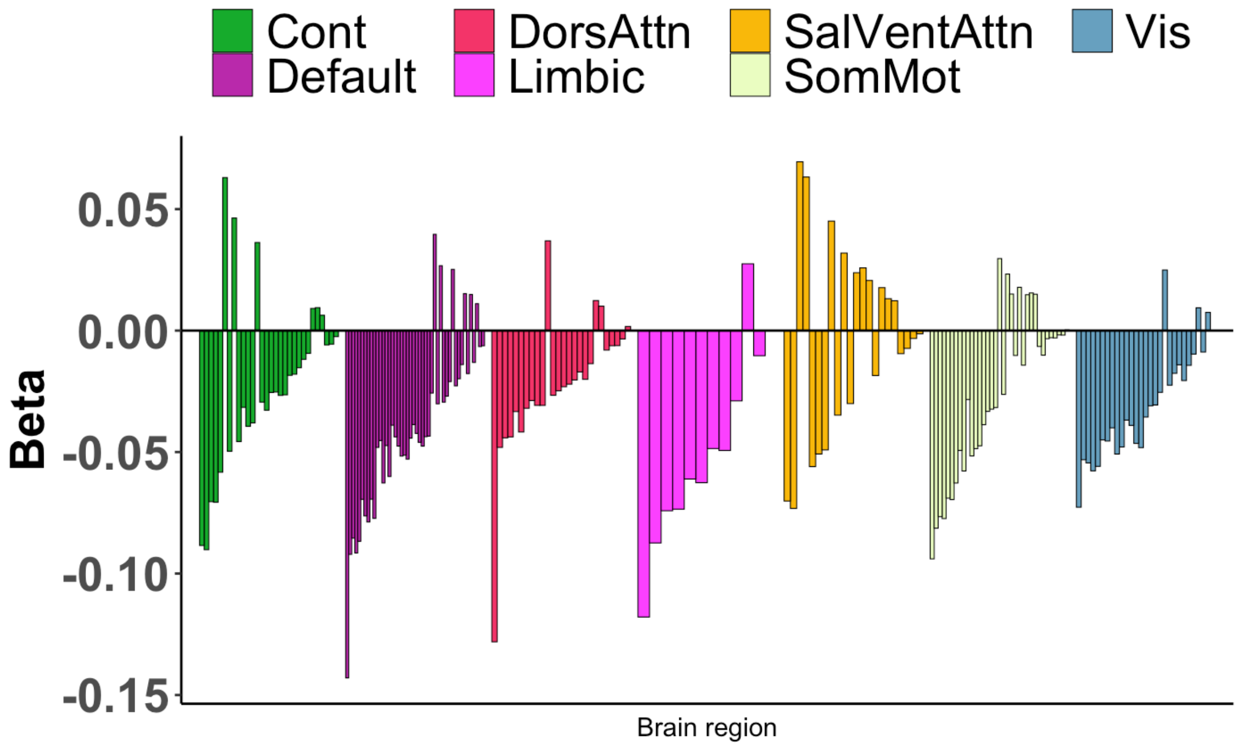

Supplementary Figure 14. Pre-existing neural similarity across brain regions spanning multiple brain networks showed a trending negative association with change in social distance over time. Regression coefficients for each brain parcel from the linear mixed model examining the relationship between pre-existing neural similarity and continuous change in social distance, grouped by their corresponding brain networks, are shown.

Supplementary Table 1. Summary of video clips shown in the fMRI study

**Clip**		**Description**	**Duration (s)**
1	‘An Astronaut’s View of Earth’	An astronaut discusses viewing Earth from space, and in particular, witnessing the effects of climate change from space. He then urges viewers to mobilize to address this issue.	223
2	Google Glass review	A journalist wears a Google Glass headset for a day and weighs the pros and cons of being an ‘early adopter’ of this technology.	88
3	‘Crossfire’	Two journalists debate the appropriateness of President Obama’s use of humor in a speech; excerpts from the speech are shown.	89
4	‘All I Want’	A sentimental music video depicting a social outcast with a facial deformity seeking companionship.	305
5	Wedding film	A homemade film depicting scenes from two men’s wedding ceremony and subsequent celebration with family and friends.	120
6	Scientific demonstration	An astronaut at the International Space Station demonstrates and explains what happens when one wrings out a waterlogged washcloth in space.	118
7	‘Food Inc.’	An excerpt from a documentary discussing how the fast food industry influences food production and farming practices in the United States.	178
8	‘We Can Be Heroes’	An excerpt from a mockumentary-style series in which a man discusses why he nominated himself for the title of Australian of the Year.	202
9	‘Ban College Football’	Journalists and athletes debate whether or not football should be banned as a college sport.	195
10	Soccer match	Highlights from a soccer match.	91
11	Baby sloth sanctuary	A documentary about caring for baby sloths at a sanctuary in Costa Rica.	200
12	‘Ew!’	A comedy skit in which grown men play teenage girls disgusted by things around them.	169
13	‘Life’s Too Short’	An example of ‘cringe comedy’ in which a dramatic actor is depicted unsuccessfully trying his hand at improvisational comedy.	106
14	‘America’s Funniest Home Videos’	A series of homemade video clips depicting examples of unintentional physical comedy arising from accidents.	101

Table adapted from Parkinson et al. (2018).

Supplementary Table 2. Bootstrap confidence intervals for the analysis testing if pre-existing neural similarities among individuals who grew closer over time versus who grew apart

Brain parcel	Δisc	$p_{uncorrected}$	$p_{FDR\text{-}corrected}$	CI_lower_bound	CI_higher_bound
**7Networks_LH_DorsAttn_Post_2**	0.664	<0.001	<0.001	0.236	1.040
**7Networks_LH_Vis_14**	0.929	<0.001	<0.001	0.240	1.304
**7Networks_LH_Vis_13**	0.902	<0.001	<0.001	0.290	1.269
**7Networks_RH_Vis_13**	0.796	<0.001	<0.001	0.158	1.210
**7Networks_RH_Vis_12**	0.826	<0.001	<0.001	0.131	1.205
**7Networks_RH_DorsAttn_Post_8**	0.882	<0.001	<0.001	0.143	1.341
**7Networks_RH_Vis_10**	0.818	<0.001	<0.001	0.281	1.213
**7Networks_RH_Vis_11**	0.845	<0.001	<0.001	0.074	1.262
**7Networks_RH_Vis_4**	0.954	<0.001	<0.001	0.351	1.288
**7Networks_LH_Vis_4**	0.819	<0.001	<0.001	0.242	1.180
**7Networks_LH_Cont_Cing_2**	0.477	<0.001	<0.001	0.080	0.868
**7Networks_RH_Vis_9**	0.925	<0.001	<0.001	0.378	1.269
**7Networks_RH_Vis_15**	0.917	<0.001	<0.001	0.158	1.350
**7Networks_RH_Vis_14**	0.888	0.001	0.012	0.306	1.277
**7Networks_RH_Cont_Temp_1**	0.477	0.001	0.012	0.086	0.857
**7Networks_RH_Vis_3**	0.779	0.001	0.012	0.146	1.156
**7Networks_LH_Vis_11**	0.779	0.001	0.012	0.137	1.184
**7Networks_LH_Vis_2**	0.767	0.001	0.012	0.169	1.144
**7Networks_LH_Vis_1**	0.713	0.002	0.018	0.024	1.119
**7Networks_LH_DorsAttn_Post_3**	0.750	0.002	0.018	0.099	1.172
**7Networks_LH_Vis_12**	0.678	0.002	0.018	0.061	1.076
**7Networks_LH_Vis_9**	0.709	0.002	0.018	0.161	1.053
**7Networks_RH_Cont_PFCmp_2**	0.365	0.002	0.018	-0.004	0.738
**7Networks_RH_Default_PFCm_2**	0.529	0.002	0.018	0.114	0.864
**7Networks_LH_Vis_7**	0.749	0.003	0.025	0.134	1.125
**7Networks_LH_Vis_10**	0.741	0.003	0.025	0.165	1.125
**7Networks_RH_SomMot_15**	0.419	0.004	0.030	-0.011	0.800
**7Networks_LH_Default_PFC_3**	0.389	0.004	0.030	-0.010	0.762
**7Networks_LH_Vis_3**	0.677	0.004	0.030	0.113	1.070
**R_Thalamus**	0.402	0.005	0.033	0.026	0.740
**7Networks_RH_DorsAttn_Post_9**	0.684	0.005	0.033	0.039	1.107
**7Networks_RH_Vis_7**	0.669	0.005	0.033	0.122	1.082
**L_Amygdala**	0.455	0.006	0.038	0.039	0.819
**7Networks_LH_Default_Temp_6**	0.518	0.006	0.038	0.092	0.893
**7Networks_RH_Cont_PFCl_6**	0.399	0.007	0.042	-0.017	0.761
**7Networks_RH_SomMot_17**	0.424	0.007	0.042	0.043	0.759

**7Networks_LH_Cont_PFCl_1**	0.410	0.008	0.046	-0.061	0.818
**7Networks_RH_Cont_PFCl_1**	0.377	0.009	0.048	-0.076	0.766
**7Networks_LH_Default_PFC_8**	0.455	0.009	0.048	0.008	0.893
**7Networks_RH_Default_Par_3**	0.434	0.009	0.048	-0.025	0.857
**L_Thalamus**	0.391	0.010	0.050	-0.012	0.741
**7Networks_RH_Default_PFCm_3**	0.449	0.010	0.050	-0.026	0.874
**7Networks_RH_Cont_PFCl_5**	0.410	0.010	0.050	0.005	0.772

Supplementary Table 3. Bootstrap confidence intervals for the analysis testing if pre-existing neural similarities among individuals who grew closer over time versus who grew apart when controlling for demographic variables

Brain parcel	Δisc	$p_{uncorrected}$	$p_{FDR\text{-}corrected}$	CI_lower_bound	CI_higher_bound
**7Networks_RH_Vis_9**	0.823	<0.001	<0.001	0.253	1.138
**7Networks_RH_Vis_11**	0.774	<0.001	<0.001	-0.010	1.205
**7Networks_RH_Vis_15**	0.849	<0.001	<0.001	0.098	1.277
**7Networks_LH_DorsAttn_Post_2**	0.705	<0.001	<0.001	0.258	1.068
**7Networks_LH_Cont_Cing_2**	0.481	<0.001	<0.001	0.098	0.859
**7Networks_LH_Vis_13**	0.868	<0.001	<0.001	0.243	1.218
**7Networks_RH_Vis_4**	0.845	<0.001	<0.001	0.251	1.180
**7Networks_LH_Vis_14**	0.880	0.001	0.015	0.222	1.228
**7Networks_RH_Cont_Temp_1**	0.463	0.001	0.015	0.070	0.814
**7Networks_LH_Vis_9**	0.722	0.001	0.015	0.198	1.018
**7Networks_LH_Vis_11**	0.767	0.001	0.015	0.165	1.150
**7Networks_LH_Vis_4**	0.744	0.001	0.015	0.137	1.101
**7Networks_RH_Vis_12**	0.801	0.001	0.015	0.100	1.140
**7Networks_RH_DorsAttn_Post_8**	0.847	0.001	0.015	0.123	1.268
**7Networks_RH_Vis_13**	0.699	0.002	0.023	0.043	1.099
**7Networks_RH_Cont_PFCmp_2**	0.410	0.002	0.023	0.049	0.743
**7Networks_RH_Default_PFCm_2**	0.476	0.002	0.023	0.073	0.774
**7Networks_RH_Vis_3**	0.734	0.002	0.023	0.139	1.091
**7Networks_RH_Vis_10**	0.715	0.002	0.023	0.136	1.093
**7Networks_LH_DorsAttn_Post_3**	0.733	0.003	0.027	0.104	1.138
**7Networks_LH_Vis_10**	0.662	0.003	0.027	0.081	1.054
**7Networks_RH_Vis_14**	0.804	0.003	0.027	0.169	1.182
**7Networks_LH_Default_Temp_6**	0.549	0.003	0.027	0.129	0.911
**7Networks_LH_Vis_3**	0.686	0.003	0.027	0.110	1.051
**7Networks_LH_Vis_2**	0.690	0.004	0.030	0.078	1.066
**7Networks_LH_Default_PFC_3**	0.384	0.004	0.030	-0.003	0.742
**7Networks_RH_Cont_PFCl_6**	0.428	0.004	0.030	0.028	0.776
**7Networks_LH_Vis_12**	0.597	0.004	0.030	-0.047	0.978
**7Networks_LH_Vis_7**	0.727	0.004	0.030	0.123	1.088
**7Networks_LH_DorsAttn_Post_7**	0.683	0.006	0.043	0.011	1.105
**7Networks_LH_Vis_1**	0.644	0.007	0.043	-0.014	1.068
**7Networks_RH_Cont_PFCl_1**	0.352	0.007	0.043	-0.114	0.732
**7Networks_RH_Default_PFCm_3**	0.415	0.007	0.043	-0.041	0.786
**7Networks_RH_DorsAttn_Post_9**	0.658	0.007	0.043	0.010	1.062
**7Networks_LH_Cont_PFCl_1**	0.377	0.007	0.043	-0.082	0.744
**7Networks_LH_Vis_8**	0.612	0.008	0.044	0.014	1.027

**R_Thalamus**	0.351	0.008	0.044	-0.044	0.710
**7Networks_RH_Cont_PFCl_5**	0.412	0.008	0.044	0.007	0.754
**7Networks_RH_Default_Par_3**	0.452	0.008	0.044	-0.007	0.849

Supplementary Table 4. Bootstrap confidence intervals for the analysis testing if pre-existing neural similarities among individuals who grew closer over time versus who grew apart when excluding dyads with self-reported interactions prior to the neuroimaging session

Brain parcel	Δisc	$p_{uncorrected}$	$p_{FDR\text{-}corrected}$	CI_lower_bound	CI_higher_bound
**7Networks_LH_DorsAttn_Post_2**	0.674	<0.001	<0.001	0.239	1.053
**7Networks_RH_DorsAttn_Post_8**	0.892	<0.001	<0.001	0.124	1.343
**7Networks_LH_Vis_2**	0.774	<0.001	<0.001	0.141	1.168
**7Networks_LH_Cont_Cing_2**	0.458	<0.001	<0.001	0.068	0.852
**7Networks_LH_Vis_4**	0.814	<0.001	<0.001	0.222	1.175
**7Networks_RH_Vis_3**	0.783	<0.001	<0.001	0.133	1.170
**7Networks_RH_Vis_4**	0.953	<0.001	<0.001	0.342	1.297
**7Networks_RH_Vis_9**	0.914	<0.001	<0.001	0.317	1.266
**7Networks_RH_Vis_10**	0.819	<0.001	<0.001	0.263	1.221
**7Networks_RH_Vis_15**	0.916	<0.001	<0.001	0.107	1.355
**7Networks_RH_Vis_12**	0.817	<0.001	<0.001	0.098	1.208
**7Networks_RH_Vis_13**	0.802	<0.001	<0.001	0.142	1.230
**7Networks_LH_Vis_13**	0.905	<0.001	<0.001	0.258	1.282
**7Networks_LH_Vis_14**	0.938	<0.001	<0.001	0.232	1.319
**7Networks_RH_Vis_11**	0.837	<0.001	<0.001	0.040	1.257
**7Networks_LH_Vis_11**	0.783	0.001	0.013	0.117	1.192
**7Networks_RH_Vis_14**	0.887	0.001	0.013	0.298	1.280
**7Networks_RH_Default_PFCm_2**	0.511	0.002	0.019	0.102	0.853
**7Networks_LH_Vis_9**	0.708	0.002	0.019	0.141	1.059
**7Networks_LH_Vis_1**	0.723	0.002	0.019	0.027	1.129
**7Networks_LH_DorsAttn_Post_3**	0.752	0.002	0.019	0.074	1.170
**7Networks_LH_Vis_12**	0.689	0.002	0.019	0.052	1.098
**7Networks_RH_Cont_Temp_1**	0.473	0.003	0.025	0.068	0.863
**7Networks_LH_Vis_10**	0.738	0.003	0.025	0.126	1.125
**7Networks_LH_Vis_7**	0.757	0.003	0.025	0.132	1.134
**7Networks_LH_Vis_3**	0.679	0.003	0.025	0.094	1.081
**7Networks_LH_Default_Temp_6**	0.543	0.004	0.031	0.138	0.910
**7Networks_LH_Default_PFC_3**	0.416	0.004	0.031	0.006	0.786
**R_Thalamus**	0.402	0.005	0.036	0.012	0.746
**7Networks_RH_DorsAttn_Post_9**	0.667	0.005	0.036	0.000	1.091
**7Networks_RH_Cont_PFCl_6**	0.408	0.006	0.039	-0.013	0.765
**7Networks_RH_Cont_PFCmp_2**	0.343	0.006	0.039	-0.030	0.724
**7Networks_RH_Vis_7**	0.672	0.006	0.039	0.118	1.085
**L_Amygdala**	0.430	0.007	0.044	0.014	0.793
**7Networks_LH_Cont_PFCl_1**	0.425	0.008	0.048	-0.062	0.832

**7Networks_RH_SomMot_17**	0.414	0.008	0.048	0.033	0.759

Supplementary Table 5. Bootstrap confidence intervals for the analysis testing if pre-existing neural similarities among individuals who grew closer over time versus who grew apart when controlling for enjoyment and interest ratings

Brain parcel	Δisc	$p_{uncorrected}$	$p_{FDR\text{-}corrected}$	CI_lower_bound	CI_higher_bound
**7Networks_LH_Vis_2**	0.785	<0.001	<0.001	0.182	1.160
**7Networks_LH_Cont_Cing_2**	0.481	<0.001	<0.001	0.104	0.863
**7Networks_LH_Vis_4**	0.839	<0.001	<0.001	0.241	1.205
**7Networks_RH_Vis_13**	0.820	<0.001	<0.001	0.162	1.239
**7Networks_RH_Vis_4**	0.972	<0.001	<0.001	0.365	1.316
**7Networks_RH_DorsAttn_Post_8**	0.890	<0.001	<0.001	0.132	1.329
**7Networks_RH_Vis_9**	0.937	<0.001	<0.001	0.371	1.287
**7Networks_RH_Vis_10**	0.838	<0.001	<0.001	0.280	1.261
**7Networks_RH_Vis_15**	0.918	<0.001	<0.001	0.145	1.354
**7Networks_LH_DorsAttn_Post_2**	0.669	<0.001	<0.001	0.241	1.041
**7Networks_LH_Vis_13**	0.920	<0.001	<0.001	0.290	1.276
**7Networks_LH_Vis_14**	0.935	<0.001	<0.001	0.254	1.296
**7Networks_RH_Vis_11**	0.855	<0.001	<0.001	0.065	1.282
**7Networks_RH_Vis_14**	0.905	0.001	0.011	0.301	1.295
**7Networks_RH_Vis_3**	0.797	0.001	0.011	0.168	1.190
**7Networks_LH_Vis_12**	0.714	0.001	0.011	0.097	1.106
**7Networks_RH_Cont_Temp_1**	0.495	0.001	0.011	0.110	0.860
**7Networks_RH_Vis_12**	0.837	0.001	0.011	0.122	1.205
**7Networks_LH_Vis_11**	0.792	0.001	0.011	0.136	1.186
**7Networks_LH_Vis_9**	0.714	0.002	0.019	0.168	1.051
**7Networks_LH_Vis_7**	0.764	0.002	0.019	0.160	1.131
**7Networks_RH_Cont_PFCmp_2**	0.378	0.002	0.019	0.021	0.747
**7Networks_LH_DorsAttn_Post_3**	0.754	0.003	0.025	0.110	1.172
**7Networks_LH_Vis_1**	0.729	0.003	0.025	0.033	1.130
**7Networks_LH_Vis_10**	0.756	0.003	0.025	0.162	1.153
**7Networks_RH_Default_PFCm_2**	0.541	0.003	0.025	0.134	0.874
**7Networks_LH_Vis_3**	0.674	0.004	0.031	0.099	1.064
**7Networks_RH_DorsAttn_Post_9**	0.707	0.004	0.031	0.059	1.122
**7Networks_RH_Cont_PFCl_6**	0.416	0.005	0.031	0.005	0.764
**7Networks_RH_Default_Par_3**	0.461	0.005	0.031	-0.001	0.848
**R_Thalamus**	0.399	0.005	0.031	0.020	0.730
**7Networks_LH_Default_PFC_3**	0.395	0.005	0.031	0.001	0.760
**7Networks_RH_SomMot_15**	0.416	0.005	0.031	-0.030	0.791
**7Networks_RH_Vis_7**	0.685	0.005	0.031	0.141	1.099
**7Networks_LH_Cont_PFCl_1**	0.416	0.006	0.036	-0.053	0.817

**7Networks_LH_Default_Temp_6**	0.515	0.006	0.036	0.092	0.882
**L_Amygdala**	0.457	0.007	0.038	0.059	0.811
**7Networks_RH_Cont_PFCl_1**	0.404	0.007	0.038	-0.058	0.782
**7Networks_RH_Cont_PFCl_5**	0.426	0.007	0.038	0.016	0.762
**7Networks_LH_Default_PFC_8**	0.468	0.008	0.042	0.003	0.882
**7Networks_RH_Limbic_OFC_2**	0.380	0.008	0.042	0.000	0.695
**7Networks_RH_Default_PFCm_3**	0.461	0.009	0.046	0.000	0.871
**7Networks_RH_DorsAttn_Post_6**	0.621	0.010	0.049	-0.100	1.085
**L_Thalamus**	0.389	0.010	0.049	0.006	0.734

Supplementary Information on Recruitment and Data Collection

Given the high response rates for both waves of the social network data and in response to one reviewer's concern on ethical consideration, here we provide more context on the collection of the social network data. The social network data were collected as part of the course pedagogy on social networks, which are integral to success in business and, therefore, a part of the core course in organizational behavior. These social network survey data are collected for every cohort of this business school, and the current fMRI study recruited participants from a cohort in which everyone participated in the social network survey, but there have been students from other cohorts who have declined to complete the survey with no formal or informal consequences. The survey was set up by the course instructor as an optional class exercise and all data were collected using student ID numbers, which were later deidentified. Personalized feedback reports were provided by the instructor of the course for each student who completed the survey. As every email account at the university has one email address based on the student's name and another email address based on their student ID number, the personalized report was sent to the email address based on their ID number. The class later discussed the overall network structure of the business school community, to which everyone taking the course belonged and could relate, whether or not they had completed the survey.

The personalized feedback report started with the following paragraphs:

"Below, please find your personalized report on the structure of your network, how it has changed since the fall, and how it compares to those of your classmates. You'll also find a diagram of your immediate network – you and the people directly tied to you.

As you interpret these results, please remember that there are no answers that are either good or bad. Your network is a means to achieving your social and professional goals, so the ideal network for you is whatever network will help you to do that. While these data describe your socializing network among your classmates, research shows that people tend to exhibit the same network-building tendencies across domains – so your professional network may bear some similarity in structure to your socializing network.

The purpose of this exercise is to give you feedback that will help you to treat your social capital as a resource worth investing in, as discussed in Personal Leadership. If you look at these results and find them to be consistent with your prior beliefs, then you have a realistic sense of your network. If, however, you find one or more of the results surprising, it may be worth thinking about how you might work more effectively to build the network you want to have."

Following the introduction, the report included definitions of key network characteristics (e.g., size, churn, diversity, and brokerage), the student's personal network characteristics as well as how their metrics compared to the average of their cohort, and a network diagram. Anecdotally, students have found the information in the report interesting and valuable. In addition, the data was collected in a small business school located in a remote part of New Hampshire, and the culture of the school is known to be extremely friendly and collegial, which may in part contribute to the high response rate.

The experimental procedures were in compliance with the IRB of the institution, and some additional evidence may help mitigate any potential concerns surrounding ethical considerations. First, it is very uncommon for faculty members to write recommendations for MBA students, as such letters are not prioritized by employers in their evaluations for potential hires. Further, a grade non-disclosure policy, which forbids students from disclosing their grades to prospective employers under penalty of the school's Honor Code, was implemented to promote collaboration among students. As a result, students at this institution have little incentive to be concerned about their grades. This policy is well-established at this business school and several peer institutions' business schools such that students and employers are aware of the policy and understand the implication behind the policy that a student's success in the program is not measured by their grades.

Supplementary Analyses Using Linear Mixed Models Rather Than Node-Level Permutation Testing

To complement our main analyses, which used permutation testing to account for non-independence in the data, we also ran multilevel linear mixed-effects models with crossed random effects for participants. For our first set of analyses, which tested if pre-existing neural similarity is higher among friends (i.e., dyads characterized by a social distance of 1) than people who were more socially distant in the network (i.e., social distance of 2 and/or more), we followed the methods outlined in Chen et al. (2017)[1], which accounted for the non-independence in the data as a result of each participant appearing in multiple dyads. Specifically, we doubled the data to add redundancy in order to allow fully crossed random effects in the linear mixed-effects model, and prior to determining statistical significance, corrected for degrees of freedom to $N - k$ (i.e., N is the number of unique dyads and k is the number of fixed effects in the model). Then, for each of the 214 brain regions, we used the R packages lmerTest 3.1-3[2] and lme4 1.1-34[2] to fit linear mixed-effects models with random intercepts for both members of the dyad to assess if neural similarity varied as a function of future levels of social distance. To compare neural similarity between dyads of different levels of social distance (e.g., $ISC_{\text{dyads that have social distance of 1}}$ vs $ISC_{\text{dyads that have social distance of 3}}$), we ran planned contrast analyses using EMMEANS 1.8.7[3] in R. We FDR-corrected all p-values and report results below that survive a significance threshold of $p < 0.05$, corrected for multiple comparisons.

For our first set of analyses testing if friends had greater pre-existing neural similarity than people who ended up farther removed from each other in the social network, we found convergent results with those of our main analyses. More specifically, pre-existing similarity in the left OFC significantly was significantly greater for future friends (i.e., dyads at a social distance of 1 from one another in the network) than for dyads who were characterized by a social distance of 3 ($\beta = 0.47$, SE = 0.12, $p < 0.001$, FDR-corrected; see Supplementary Fig. 12).

For our second set of analyses testing if pre-existing neural similarity would vary as a function of the direction of future changes in social distance in the network, we followed a similar procedure to that outlined above to run another linear mixed-effects model with crossed random effects for participants, followed by a planned contrast analysis (i.e., $ISC_{\text{dyads that grew closer}}$ vs $ISC_{\text{dyads that grew apart}}$; $ISC_{\text{dyads that grew closer}}$ vs $ISC_{\text{dyads that have no change in their social distance}}$; $ISC_{\text{dyads that grew apart}}$ vs $ISC_{\text{dyads that have no change in their social distance}}$). For the contrast $ISC_{\text{dyads that grew closer vs}}$ $ISC_{\text{dyads that grew apart}}$), although none of the brain parcels reached significance after correcting for multiple tests, we observed a similar pattern of results to that observed in our main analyses, such that dyads that grew closer generally tended to show higher pre-existing neural similarity than those that grew farther apart across a wide range of brain regions (see Supplementary Fig. 13).

We also ran analyses examining social distance change as a continuous variable in a linear mixed-effects model with crossed random effects for participants using a similar approach as described above. We found that pre-existing neural similarity in regions of the ventromedial prefrontal cortex and lateral temporal cortex significantly were linked to future decreases in social distance over time (see Supplementary Figs. 14 and 15).

References

1. Chen, G., Taylor, P. A., Shin, Y. W., Reynolds, R. C. & Cox, R. W. Untangling the relatedness among correlations, Part II: Inter-subject correlation group analysis through linear mixed-effects modeling. *NeuroImage*, **147**, 825–840 (2017).

2. Kuznetsova, A., Brockhoff, P. B., & Christensen, R. H. B. lmerTest Package: Tests in linear mixed effects models. *J. Stat. Softw.* **82**(13), 1–26 (2017).

3. Lenth, R. emmeans: Estimated marginal means, aka least-squares means. R package version 1.8.7 (2023).
