# Supplementary material for: Neural similarity predicts whether strangers become friends
Source: Nat Hum Behav. 2025 Aug 4;9(11):2285–98. doi: 10.1038/s41562-025-02266-7 (PMC12634445; doi:10.1038/s41562-025-02266-7)
Supplement: Supplementary file 2 — Reporting Summary [file 41562_2025_2266_MOESM2_ESM.pdf]

nature portfolio

Corresponding author(s): Carolyn Parkinson

Last updated by author(s): 3/18/2025

Reporting Summary

Nature Portfolio wishes to improve the reproducibility of the work that we publish. This form provides structure for consistency and transparency in reporting. For further information on Nature Portfolio policies, see our Editorial Policies and the Editorial Policy Checklist.

Statistics

For all statistical analyses, confirm that the following items are present in the figure legend, table legend, main text, or Methods section.

n/a	Confirmed	
☐	☒	The exact sample size (*n*) for each experimental group/condition, given as a discrete number and unit of measurement
☐	☒	A statement on whether measurements were taken from distinct samples or whether the same sample was measured repeatedly
☐	☒	The statistical test(s) used AND whether they are one- or two-sided<br>*Only common tests should be described solely by name; describe more complex techniques in the Methods section.*
☐	☒	A description of all covariates tested
☐	☒	A description of any assumptions or corrections, such as tests of normality and adjustment for multiple comparisons
☐	☒	A full description of the statistical parameters including central tendency (e.g. means) or other basic estimates (e.g. regression coefficient) AND variation (e.g. standard deviation) or associated estimates of uncertainty (e.g. confidence intervals)
☐	☒	For null hypothesis testing, the test statistic (e.g. F, t, r) with confidence intervals, effect sizes, degrees of freedom and P value noted<br>*Give P values as exact values whenever suitable.*
☒	☐	For Bayesian analysis, information on the choice of priors and Markov chain Monte Carlo settings
☐	☒	For hierarchical and complex designs, identification of the appropriate level for tests and full reporting of outcomes
☐	☒	Estimates of effect sizes (e.g. Cohen's *d*, Pearson's *r*), indicating how they were calculated

Our web collection on statistics for biologists contains articles on many of the points above.

Software and code

Policy information about availability of computer code

Data collection	Participants were scanned using a Siemens Prisma 3T scanner.
Data analysis	We used fMRIPrep version 1.4.0 to preprocess our fMRI data. We calculated inter-subject correlations (ISCs) using SciPy, normalized the ISCs using Scikit-learn 0.24.2 and permuted ISCs at subject-level in Python 3.9.6. The social network was characterized using igraph 1.5.1 in Python. Statistical tests (i.e., permutation tests) were conducted in Python 3.9.6. Custom codes developed for the study is available at https://github.com/lisashen-syx/Neural-Similarity-Predicts-Whether-Strangers-Become-Friends.git

For manuscripts utilizing custom algorithms or software that are central to the research but not yet described in published literature, software must be made available to editors and reviewers. We strongly encourage code deposition in a community repository (e.g. GitHub). See the Nature Portfolio guidelines for submitting code & software for further information.

Data

Policy information about availability of data

All manuscripts must include a data availability statement. This statement should provide the following information, where applicable:

- Accession codes, unique identifiers, or web links for publicly available datasets
- A description of any restrictions on data availability
- For clinical datasets or third party data, please ensure that the statement adheres to our policy

Source data for this study (specifically, the inter-subject correlation and social distance data that we generated in this study, as well as dyadic dissimilarities in

demographic variables and dyadic dissimilarities in ratings of enjoyment of and interest in the stimuli) are available at https://github.com/lisashen-syx/Neural-Similarity-Predicts-Whether-Strangers-Become-Friends.git

Research involving human participants, their data, or biological material

Policy information about studies with human participants or human data. See also policy information about sex, gender (identity/presentation), and sexual orientation and race, ethnicity and racism.

Reporting on sex and gender	As part of the social network survey, participants self-reported their gender. As our research question of interest does not concern friendship formation or social network based on gender, gender-based analyses were not performed as part of our primary analyses. Gender was included as a control variable in a set of analyses controlling for sociodemographic variables in which the inter-subject similarities in such variables (e.g., age, gender, nationality) were regressed out from ISCs in each brain region before repeating the methods used in the main analyses. Given that the analyses of our study were conducted on dyad-level data, we have only included a binary variable "gender_similarity" to indicate whether two people in the dyad self-report the same gender or not. Thus, individual-level data on gender was not shared.
Reporting on race, ethnicity, or other socially relevant groupings	Participants who completed the social network survey also self-reported their nationality, which is defined as the nation where they hold citizenship. We conducted additional analyses controlling for sociodemographic variables (e.g., age, gender, nationality, size and location of hometown, location and type of undergraduate institution, industry of employment) by regressing out inter-subject similarities in these variables from ISCs in each brain region before repeating the methods used in the main analyses.
Population characteristics	The research sample consists of a first-year graduate student (M.B.A) cohort at a private university in the United States (N = 288; 129 female; Mean age = 28.5 years). A subset of 43 individuals (14 female; Mean age= 28.6) self-selected to participate in the fMRI study after receiving a recruitment email that was sent to all incoming students in the cohort. Of the 43 participants, one participant did not complete the scan and was excluded from analysis and one participant did not participate in the social network survey administered at Time 3 (see Social network survey participants) and thus was excluded from analysis. Data from the resulting 41 participants (14 female) aged 25-34 (M = 28.63, SD = 2.15) were used for analysis. Of these 41 participants, 38 were right-handed and three were left-handed. Twenty-four participants self-identified their nationality as that of the United States, with five participants from India, two participants from Australia, two participants from Peru, and one participant each from Argentina, Iran, Russia, Kyrgyzstan, the United Kingdom, China, Brazil, and Canada. Fifteen participants identified as White/Non-Hispanic, two identified as Hispanic/Latino, one identified as Black/Non-Hispanic, one identified as Asian/Asian-American/Pacific-Islander, one identified as multi-racial, and 21 chose to not indicate their ethnicity.
Recruitment	Social network survey: All students in the first-year graduate cohort enrolled in a leadership course within the graduate program were invited to participate in the online social network survey as an optional part of their coursework on leadership. They were reminded that participation was voluntary and would not affect their performance in the course.<br>fMRI study: A subset of 43 individuals from an incoming graduate student (M.B.A.) cohort (Ncohort = 288) at a private university in the United States participated in the neuroimaging study. Participants self-selected to participate in the study after receiving a recruitment email that was sent to all incoming students in the cohort.
Ethics oversight	Institutional Review Board of Dartmouth College

Note that full information on the approval of the study protocol must also be provided in the manuscript.

Field-specific reporting

Please select the one below that is the best fit for your research. If you are not sure, read the appropriate sections before making your selection.

☐ Life sciences ☒ Behavioural & social sciences ☐ Ecological, evolutionary & environmental sciences

For a reference copy of the document with all sections, see nature.com/documents/nr-reporting-summary-flat.pdf

Behavioural & social sciences study design

All studies must disclose on these points even when the disclosure is negative.

Study description	This is a quantitative study involving tests for associations between fMRI and longitudinal social network data.
Research sample	The research sample consists of a first-year graduate student (M.B.A) cohort at a private university in the United States (N = 288; 129 female; Mean age = 28.5 years). A subset of 43 individuals (14 female; Mean age = 28.6) self-selected to participate in the fMRI study after receiving a recruitment email that was sent to all incoming students in the cohort. As the aim of the study involves investigating whether pre-existing neural similarities can predict future social network structure (e.g., whether two people befriend one another or grew closer over time) and thus characterizing the social network over time, we sought to recruit individuals who have opportunities to interact frequently and form connections with one another within a relatively bounded community, where variation in the relationships among individuals may be observed. Therefore, although this sample may not be representative of the general population at this age, deliberate choice to recruit from this graduate cohort is appropriate for research question of interest.
Sampling strategy	The sampling procedure is convenience sampling based on their willingness to participate. The neuroimaging study was advertised to all students in the cohort via email, and all students who were interested in participating and who passed a standard MRI safety

	screening participated in the scan. No sample size calculation was performed as we sought to recruit as many participants in the first-year graduate student cohort as possible to be able to characterize the social network accurately and to study our research question of interest.
Data collection	The social network survey was administered online and participants completed it privately in the location of their choice. fMRI data was collected using a 3T Siemens Prisma scanner at a private university in the Untied States. Participants provided informed consent in accordance with the policies of the institution's ethical review board. No one was present besides the participant and the researchers, and the researchers were not blind to the study hypothesis during fMRI data collection, but they had no knowledge of the participants' social network data as these data were collected later.
Timing	[redacted]
Data exclusions	Of the 43 fMRI participants, one participant did not complete the scan and was excluded from analysis and one participant did not participate in the social network survey administered at Time 3 (see Social network survey participants) and thus was excluded from analysis.
Non-participation	Of the 43 fMRI participants, one participant did not complete the scan and one participant did not participate in the social network survey administered at Time 3.
Randomization	Participants were not allocated into experimental groups by the experimenters. That said, students were subject to substantial randomization with respect to their interaction opportunities with other members of the cohort. Specifically, students were randomly assigned to study groups using stratified random sampling, such that students completed all of their coursework with the same group of randomly assigned classmates in each term. Additionally, nearly all students applied to on-campus housing and were randomly assigned to housing units based on a lottery system

Reporting for specific materials, systems and methods

We require information from authors about some types of materials, experimental systems and methods used in many studies. Here, indicate whether each material, system or method listed is relevant to your study. If you are not sure if a list item applies to your research, read the appropriate section before selecting a response.

Materials & experimental systems

n/a	Involved in the study
☒	☐ Antibodies
☒	☐ Eukaryotic cell lines
☒	☐ Palaeontology and archaeology
☒	☐ Animals and other organisms
☒	☐ Clinical data
☒	☐ Dual use research of concern
☒	☐ Plants

Methods

n/a	Involved in the study
☒	☐ ChIP-seq
☒	☐ Flow cytometry
☐	☒ MRI-based neuroimaging

Plants

Seed stocks	N/A
Novel plant genotypes	N/A
Authentication	N/A

Magnetic resonance imaging

Experimental design

Design type	Naturalistic movie-watching task
Design specifications	Six functional runs (ranging from 104 TRs to 272 TRs) per session and subject
Behavioral performance measures	No behavioral performance was measured during the scans

Acquisition

Imaging type(s)	structural and functional MRI
Field strength	3T
Sequence & imaging parameters	Functional scans: spin echo; EPI sequence; 25 ms echo time (TE); 2000 ms repetition time (TR); 3.0 mm x 3.0 mm 3.0 mm resolution; 240 mm FOV; 40 interleaved transverse slices with no gap<br>T1-weighted anatomical scan: 2.32 ms TE; 2300 ms TR; 240 mm FOV; 0.9 mm x 0.9 mm x 0.9 mm resolution
Area of acquisition	A whole brain scan was used.

Diffusion MRI ☐ Used ☒ Not used

Preprocessing

Preprocessing software	fMRIPrep version 1.4.0
Normalization	The T1-weighted (T1w) image was corrected for intensity non-uniformity (INU) with N4BiasFieldCorrection, distributed with ANTs 2.1.0, and used as T1w-reference throughout the workflow. The T1w-reference was then skull-stripped with a Nipype implementation of the antsBrainExtraction.sh workflow (from ANTs), using OASIS30ANT as target template. Brain tissue segmentation of cerebrospinal fluid (CSF), white-matter (WM) and gray-matter (GM) was performed on the brain-extracted T1w using FSL FAST. Volume-based spatial normalization to MNI152NLin2009cAsym standard space was performed through nonlinear registration with antsRegistration (ANTs 2.1.0), using brain-extracted versions of both T1w reference and the T1w template.
Normalization template	MNI152NLin2009cAsym standard space (ICBM 152 Nonlinear Asymmetrical template version 2009c)
Noise and artifact removal	Automatic removal of motion artifacts using independent component analysis (ICA-AROMA) was performed on the preprocessed BOLD on MNI space time-series after removal of non-steady state volumes and spatial smoothing with an isotropic, Gaussian kernel of 6mm FWHM (full-width half-maximum). The confounding variables generated by fMRIPrep that were used as nuisance variables in the current study included global signals extracted from the CSF, WM, and whole-brain masks, framewise displacement, three translational motion parameters, and three rotational motion parameters. These confounds were regressed out of the data for each preprocessed run. Temporal filtering was performed with a band-pass filter between 0.009 and 0.08 Hz.
Volume censoring	N/A

Statistical modeling & inference

Model type and settings	We calculated inter-subject correlations (ISCs) of fMRI time series of neural responses to capture similarity in neural responses across subjects during the processing of naturalistic stimuli. First, we extracted the mean-response time series across the video-viewing task from (1) each of the 200 cortical parcels in the 200-parcel version of the Schaefer et al. (2018) parcellation scheme and (2) 14 subcortical parcels in the Harvard-Oxford subcortical atlas, which resulted in a total of 214 brain regions across the whole brain. For each unique pair of participants (i.e., dyads) in our fMRI sample, we computed the Pearson correlation between the dyad members' time series of neural responses for each cortical parcel. This yields one correlation coefficient per unique dyad for each brain parcel. We then used a permutation testing procedure to test whether the mean neural similarity differed between levels of social distance at Time 3 and direction of change in social distance from Time 2 to Time 3.
Effect(s) tested	We tested (1) if pre-existing neural similarity at Time 1 differed between levels of social distance at Time 3 and (2) if pre-existing neural similarity at Time 1 significantly differed as a function of the direction of change in social distance between Time 2 and Time 3.

Specify type of analysis: ☒ Whole brain ☐ ROI-based ☐ Both

Statistic type for inference<br>(See Eklund et al. 2016)	As described in the "Model type and settings" field above, our analyses used responses within each of 214 anatomically-defined brain regions, and thus are not impacted by the concerns that the Eklund et al. (2016) paper raised regarding inflated false-positive rates in fMRI inferences for spatial extent.<br><br>We used False-Discovery Rate (FDR) correction to correct for multiple comparisons across brain regions.
Correction	We used FDR correction for multiple comparisons.

Models & analysis

n/a	Involved in the study
☒	☐ Functional and/or effective connectivity
☒	☐ Graph analysis
☒	☐ Multivariate modeling or predictive analysis
